# Isopropyl Gallate, a Gallic Acid Derivative: In Silico and In Vitro Investigation of Its Effects on *Leishmania major*

**DOI:** 10.3390/pharmaceutics14122701

**Published:** 2022-12-02

**Authors:** Danielly Silva de Melo, José Arimatéa de Oliveira Nery Neto, Maisa de Sousa dos Santos, Vinícius Duarte Pimentel, Rita de Cássia Viana Carvalho, Valéria Carlos de Sousa, Ruy Gabriel Costa Sousa, Lázaro Gomes do Nascimento, Michel Muálem de Moraes Alves, Daniel Dias Rufino Arcanjo, Damião Pergentino de Sousa, Fernando Aécio de Amorim Carvalho

**Affiliations:** 1 Laboratory of Antileishmanial Activity (LAA), Medicinal Plants Research Center, Federal University of Piauí, Teresina 64049-550, Piauí, Brazil; 2 Laboratory of Functional and Molecular Studies in Physiopharmacology (LAFMOL), Department of Biophysics and Physiology, Federal University of Piaui, Teresina 64049-550, Piauí, Brazil; 3 Laboratory of Pharmaceutical Chemistry, Federal University of Paraíba, João Pessoa 58051-900, Paraíba, Brazil; 4 Department of Veterinary Morphophysiology, Federal University of Piauí, Teresina 64049-550, Piauí, Brazil; 5 Department of Biochemistry and Pharmacology, Federal University of Piauí, Teresina 64049-550, Piauí, Brazil

**Keywords:** polyphenols, propyl gallate, molecular docking, ADMET, antileishmania activity

## Abstract

Isopropyl gallate (IPG) is a polyphenol obtained from alterations in the gallic acid molecule via acid catalysis with previously reported leishmanicidal and trypanocidal activities. The present study aims to evaluate in silico binding activity towards some targets for antileishmanial chemotherapy against *Leishmania major* species, and ADMET parameters for IPG, as well as in vitro antileishmanial and cytotoxic effects. Molecular docking was performed using AutoDockVina and BIOVIA Discovery Studio software, whereas in silico analysis used SwissADME, *PreADMET* and admetSAR software. In vitro antileishmanial activity on promastigotes and amastigotes of *Leishmania major*, cytotoxicity and macrophages activation were assessed. IPG exhibited affinity for pteridine reductase (PTR1; −8.2 kcal/mol) and oligopeptidase B (OPB; −8.0 kcal/mol) enzymes. ADMET assays demonstrated good lipophilicity, oral bioavailability, and skin permeability, as well as non-mutagenic, non-carcinogenic properties and low risk of cardiac toxicity for IPG. Moreover, IPG inhibited the in vitro growth of promastigotes (IC_50_ = 90.813 µM), presented significant activity against amastigotes (IC_50_ = 13.45 μM), promoted low cytotoxicity in macrophages (CC_50_ = 1260 μM), and increased phagocytic capacity. These results suggest IPG is more selectively toxic to the parasite than to mammalian cells. IPG demonstrated acceptable in silico pharmacokinetics parameters, and reduced infection and infectivity in parasitized macrophages, possibly involving macrophage activation pathways and inhibition of leishmania enzymes.

## 1. Introduction

Neglected tropical diseases (NTDs) are a group of diseases caused by infectious and parasitic agents, occurring mainly in developing countries [1]. Despite representing an important public health problem, they do not attract the attention of health agencies and large pharmaceutical industries. Consequently, there is low investment in drug development, inefficient planning of low-cost and free access, besides vaccines and laboratory tests for disease diagnosis [2,3]. Among them, leishmaniasis is designated as one of the most important NTDs, according to the World Health Organization (WHO) [4]. In the Americas, leishmaniasis are present in 18 countries and the most common clinical form is cutaneous leishmaniasis (LC), while visceral leishmaniasis (VL) is the most severe form and is almost always fatal if untreated [5,6,7].

The mainstays of antileishmanial therapy are pentavalent antimonials, such as sodium stibogluconate (Pentostam) and meglumine antimoniate (Glucantime), as first-line [4] and amphotericin B (Anf B) as second-line therapy. However, even with the development of a liposomal formulation for amphotericin B, the available treatment for leishmaniasis is limited because it exhibits high toxicity, serious adverse effects, and high cost. The lack of an effective vaccine, the appearance of relapse, therapeutic failure in immunocompromised patients, and resistance to pharmacological treatment are factors that motivate the search for new drugs, among which products derived from natural sources have been widely studied [8,9]. 

An important point of exploration of natural sources are the phenolic compounds characterized by the presence of one or more aromatic rings linked to at least one hydroxyl radical and/or other substituents [10]. Gallic acid (3,4,5-trihydroxybenzoic acid) is a widely investigated phenolic compound, showing several pharmacological activities [11,12]. From gallic acid, many compounds have originated, such as the isopropyl gallate (IPG), a polyphenol obtained from alterations in the gallic acid molecule by either acid or enzymatic catalyzes. This compound has already demonstrated trypanocidal and leishmanicidal activities [13], as well as immunomodulatory and antioxidant activities underlying an anti-inflammatory effect [14].

In this context, the present study aims to evaluate the physicochemical, pharmacokinetic, and in silico toxicological properties of IPG and its antileishmanial, cytotoxic and macrophages activation potential in vitro.

## 2. Materials and Methods

### 2.1. Isopropyl Gallate Compound

**Figure 1:**
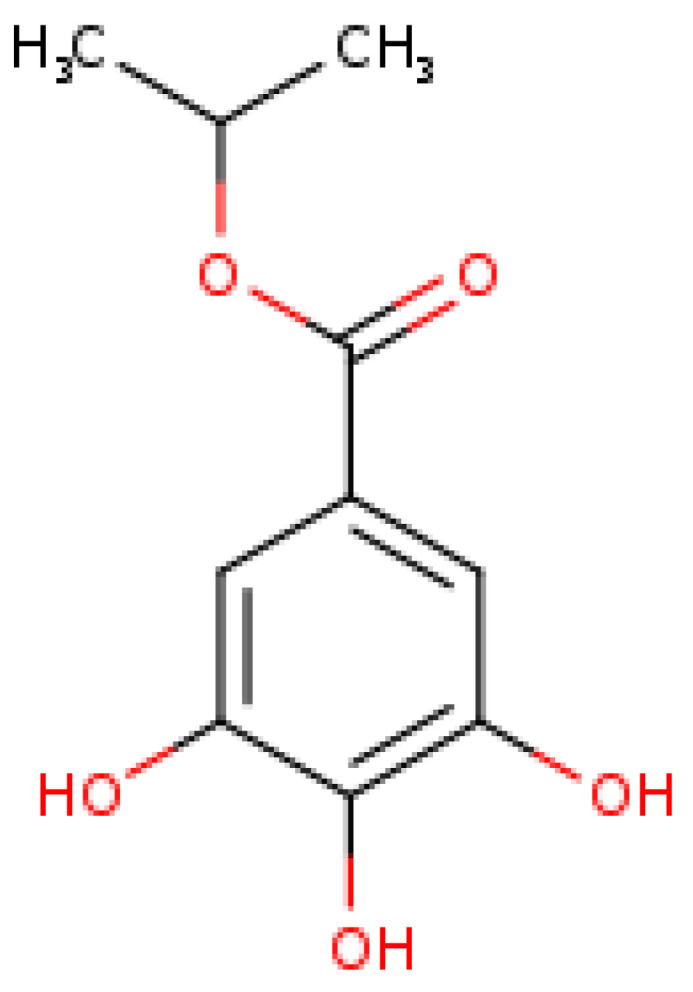
The 2D structure of IPG (isopropyl 3,4,5-trihydroxybenzoate). Molecular weight of 212.20 g/mol. Source: Swiss ADME, 2021.

Isopropyl gallate—IPG (Figure 1) was synthesized and characterized according to the literature [14]. 

### 2.2. In Silico Analysis

#### 2.2.1. Molecular Docking

Initially, the IPG was designed and its 3D structure optimized using the software ACD/ChemSketch version 14.0, based on parameters of classical mechanics (bond distance, bond angle, and dihedral angle). After optimization, the molecule was saved in (mol) format. The molecular targets used, as described in (Table 1), were obtained from the *Protein Data Bank* (PDB) and were chosen based on the literature [15], which evaluated the main targets for antileishmania chemotherapy in molecular docking studies.

Before performing the computational docking, we used the software BIOVIA Discovery Studio version 1.2.0 to remove water molecules, cofactors and possible ligands were complexed to the receptor, and then the targets were saved in (.pdb) format. Additionally, in this same program, the .mol file of the IPG ligand was also converted into .pdb format.

Finally, the ligand was imported into .pdb format in AutoDockTools 1.5.6 software, where rotational bonds were detected and defined as rigid, and polar hydrogens and Gasteiger charges were added. Then, the prepared IPG ligand was saved in .pdbqt format. The targets were kept flexible, and Gasteiger charges and polar hydrogens were also added. The non-polar hydrogens were removed and the targets saved in .pdbqt [16]. 

The volume of the docking Grid was defined at 40 points × 40 points × 40 points (X, Y and Z dimensions) for a space of 1 Å, and the center of the docking grid was defined at each target so that it encompassed the active site of the target and its adjacency. The dimensions and also coordinates in the Cartesian plane of the grid in each receiver were used to generate the AutoDock Vina configuration files. Finally, the computational docking of the IPG directed to the active site of the enzymes was performed. To evaluate the molecular interactions between the IPG and the targets, the Discovery Studio program was used, in which the images of hydrogen bonds and hydrophobic interactions were obtained [17]. 

#### 2.2.2. Analysis of Physicochemical, Pharmacokinetic and Toxicological Properties

To determine the physicochemical and pharmacokinetic properties the following tools were used; SwissADME (http://www.swissadme.ch/index.php, accessed on 22 October 2022) [18] and PreADMET (https://preadmet.bmdrc.kr/, accessed on 22 October 2022) [19]. The parameters to evaluate the physicochemical properties used were molecular weight, lipophilicity, solubility, number of rotational bonds, number of hydrogen donor and acceptor bonds, molar refractivity, and polar surface-area tension. The pharmacokinetic parameters evaluated were human oral bioavailability, human intestinal absorption, permeability coefficient in Caco-2 and in canine kidney cell model, plasma protein binding, P-glycoprotein inhibition, blood–brain barrier permeability, permeability in inhibition of cytochrome P450 enzymes, and Lipinski rule parameters.

Toxicity prediction was performed using the software admetSAR (http://lmmd.ecust.edu.cn/admetsar2, accessed on 22 October 2022) [20], evaluating organ toxicity and genomic toxicity parameters [21]. Organ toxicity was evaluated by drug-induced liver-injury assay, human gene potassium channels, acute oral toxicity, eye injury, and eye corrosion. Genomic toxicity was evaluated through ames mutagenesis, carcinogenesis, and micronucleus assay.

### 2.3. In Vitro Tests

#### 2.3.1. Parasites and Cells

*Leishmania major* promastigotes (MHOM/IL/80/Friedlin) were cultured in Schneider’s medium (Sigma^®^, St. Louis, MO, USA), supplemented with 10% fetal bovine serum (FBS) (Sigma^®^) and penicillin-streptomycin 10,000 IU/10 mg (Sigma^®^) at 26 °C in a biological oxygen demand (B.O.D) oven. 

Murine macrophages were collected from the peritoneal cavities of BALB/c mice (4 to 5 weeks old), after previous elicitation (48 h) by the application of 2 mL of 3% thioglycolate intraperitoneally. All protocols involving the use of animals were approved by the Ethics Committee on the Use of Animals from Universidade Federal do Piauí, Brazil (Protocol No. 635/20).

#### 2.3.2. Investigation of the Antileishmanial Activity of IPG on Promastigote Forms

The test was performed with promastigotes of *L. major* in their logarithmic phase of growth, in which they were seeded at 1 × 10^6^ leishmania/100 µL of medium, in 96-well cell-culture plates already containing IPG in serial dilutions, attaining eight desired final concentration ranges (29 to 3770 µM). The plate was incubated in B.O.D. at 26 °C for 48 h. When only 6 h were remaining until the end of this period, 20 µL of resazurin 1 × 10^−3^ mol/L was added, and the plate was incubated again. The plate was read on an absorbance plate reader at 550 nm, with a result in % inhibition of parasite growth. Amphotericin B (Anf B) (2.16 μM) was used as positive control, and for negative control, only Schneider’s medium containing 1 × 10^6^ promastigotes per well was used. In this case, viability was considered as 100% for the parasite [22,23].

#### 2.3.3. Cytotoxicity on Macrophages

Cytotoxicity was evaluated by the 3-[4,5-dimethylthiazol-zyl]-2,5-diphenyltetrazolium bromide (MTT) test (Sigma-Aldrich, St. Louis, MO, USA). In the 96-well plate, 2 × 10^5^ macrophages per well were incubated in 100 μL of *Roswell Park Memorial Institute* 1640 medium (RPMI 1640)-supplemented with 10% FBS and 10,000 IU penicillin and 1000 IU streptomycin in an oven at 37 °C and 5% CO_2_, for 4 h for cell adhesion. The supernatant was removed for complete elimination of non-adhered cells. Then, IPG was diluted in supplemented RPMI medium and added by serial dilutions. reaching the eight concentration ranges (29 to 3770 µM). Later, the plate was incubated in the incubator at 37 °C and 5% CO_2_ for 48 h. Subsequently, 10% MTT [5 mg/mL] was added and again the plate was incubated at 37 °C and 5% CO_2_ for 4 h. After that, the supernatant was removed and 100 μL of DMSO was added to dissolve the formazan crystals. Then, the plate was placed under stirring for about 30 min at room temperature for complete dissolution of formazan. Finally, reading was performed at 550 nm in plate reader [11].

#### 2.3.4. Cell Viability in Red Cells

Sheep red blood cells were diluted in 80 µL of PBS, adjusting the blood concentration to 5% RBC. Next, IPG was added at the desired concentrations (29 to 3770 µM), diluted in a volume of 20 µL of phosphate-buffered saline (PBS). Immediately after, they were incubated for 1 h at 37 °C and 5% CO_2_, and the reaction was stopped by the addition of 200 µL of PBS. Then, the suspensions were centrifuged at 1500 rpm at 4 °C for 10 min and the supernatants were removed and transferred to another 96-well microplate. Spectrophotometer reading was performed at 550 nm to quantify the hemolytic activity. This test was performed in triplicate, using PBS as a negative control (absence of hemolysis) and sterile Milli-Q water as a positive control (presence of hemolysis) [24]. 

#### 2.3.5. IPG Activity in L. major-Infected Macrophages

Macrophages (2 × 10^5^ cells/well) were plated on 24-well culture plates containing 13 mm sterile round coverslips and supplemented RPMI medium. The culture plates were incubated at 37 °C and 5% CO_2_ for 4 h for cell adhesion. The adhered macrophages were then incubated with a new medium containing *L. major* promastigotes (in stationary phase) at a ratio of 10 promastigote to 1 macrophage at 5% CO_2_ and 37 °C. After this period, the medium was subsequently aspirated to remove non-internalized parasites. The infected culture was incubated with the values corresponding to approximately ¼ of IC_50_ (19.00 µM) and ½ of IC_50_ (38.00 µM) values of IPG against promastigote forms, respectively. The negative control was performed with RPMI medium supplemented with 0.2% DMSO and the positive control was performed with Anf B at a concentration of 2.16 μM. After this period, the coverslips were removed and stained using Rapid Panoptic^®^. For each treatment, the number of infected macrophages and the number of internalized amastigotes were counted, covering the fields of the samples until a count of 100 macrophages was reached, using light microscopy. The results were expressed as the infection index, obtained using the following formula: % Infected macrophages × n° of amastigotes/macrophages [22].

The selectivity index (SI) was calculated by dividing the mean cytotoxic concentration (CC_50_) by the mean inhibitory concentration (IC_50_) calculated for the amastigote form of the parasite. Experiments were performed in triplicate and wells without added IPG were used as controls [25].

#### 2.3.6. Evaluation of Macrophage Activation Parameters 

##### Evaluation of Lysosomal Activity 

Peritoneal macrophages (2 × 10^5^/well) were incubated in a 96-well plate at 37 °C and 5% CO_2_ with IPG as described in Section 2.3.5. Serial dilutions were performed reaching 5 final concentration ranges (29, 58, 117, 235, 471 µM). After 48 h of incubation, 10 μL of 2% DMSO neutral red solution was added and incubated for 30 min. After that time, the supernatant was discarded, the wells were washed with 0.9% saline at 37 °C, and 100 μL of extraction solution was added to solubilize the neutral red present inside the lysosomal secretion vesicles. After 30 min under stirring, the plate was read at 550 nm [11].

##### Determination of Phagocytic Capacity 

Peritoneal macrophages were plated and incubated with IPG as described in Section 2.3.5. After 48 h of incubation, 10 μL of Zymosan solution stained with neutral red was added and incubated for 30 min at 37 °C. After this procedure, 100 μL of Baker’s fixative was added to stop the phagocytosis process and 30 min later, the plate was washed with 0.9% saline in order to remove the stained zymosan not phagocytosed by the macrophages. The supernatant was removed, and 100 μL of extraction solution was added. After solubilization under stirring, the plate was read at 550 nm [26].

##### Determination of Nitrite

Peritoneal macrophages were plated and incubated with IPG as described in Section 2.3.5. After 24 h of incubation at 37 °C and 5% CO_2_, the cell-culture supernatants were transferred to another 96-well plate for nitrite dosage. The standard curve was prepared with sodium nitrite in RPMI medium at varying concentrations of (1, 5, 10, 25, 50, 75, 100, and 150 μM) diluted in RPMI medium. At the time of dosage, equal parts of the samples (supernatants) or the solutions prepared to obtain the standard curve were mixed with the same volume of Griess reagent (1% Sulfanilamide in H_3_PO_4_ 10% (*v*/*v*) in Milli-Q^®^ water and were added in equal parts to 0.1% naphthylenediamine in Milli-Q^®^ water) and the absorbance was read at 550 nm. The result was plotted as the percentage of nitrite production [27].

### 2.4. Statistical Analysis

The results were expressed as the mean ± standard error of the mean. To evaluate the significance of the differences between the means in the in silico assay, analysis of variance (ANOVA) was used, followed by Tukey’s multiple comparisons test. The in vitro assays were performed in triplicate. The mean inhibitory concentration (IC_50_), mean cytotoxic concentration (CC_50_), and mean hemolytic concentration (CH_50_) with 95% confidence limit were calculated using probit regression. ANOVA analysis of variance followed by Bonferroni’s test was performed, taking *p* value < 0.05 as the maximum level of significance.

## 3. Results and Discussion

### 3.1. In Silico Analysis of IPG: Molecular Docking and ADMET Assessment

To determine the possible interaction of IPG with *L. major* targets, molecular docking analysis was performed with the enzymes nucleoside hydrolase (NH), oligopeptidase B (OPB), leishmanolysin proteinase (*Gp63*), pteridine reductase (PTR1), and triparedoxin peroxidase (TxP). The lowest interaction energies for each target are shown in (Table 2). The lower binding energies attest to a higher affinity of the ligand for the target, indicating that the ligand has higher attraction to the target and may become a promising molecule for clinical trials [28].

**Figure 2:**
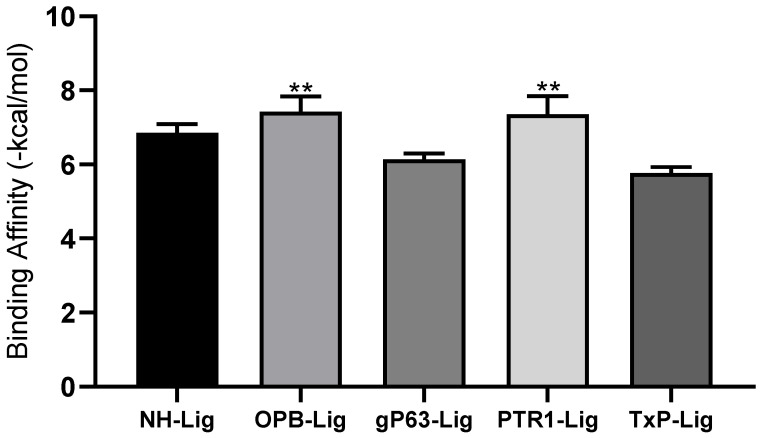
Interaction affinity between IPG and the molecular targets under study. Nucleoside hydrolase (NH); oligopeptidase B (OPB); leishmanolysin proteinase (gp63); pteridine reductase (PTR1); triparedoxin peroxidase (TxP). Two asterisks (**) Represents the molecular targets (OPB and PTR-1) that showed better binding interaction with IPG.

Binding free energy values were submitted to normality analysis using the D’Agostino and Pearson test. After confirming the normal distribution of the data, an ANOVA analysis of variance was performed followed by Tukey’s test for unpaired samples. It was observed that IPG showed a better affinity to target PTR1 (−8.2 kcal/mol) and OPB (−8.0 kcal/mol) statistically superior to NH, Gp63, and TxP (Figure 2).

**Figure 3:**
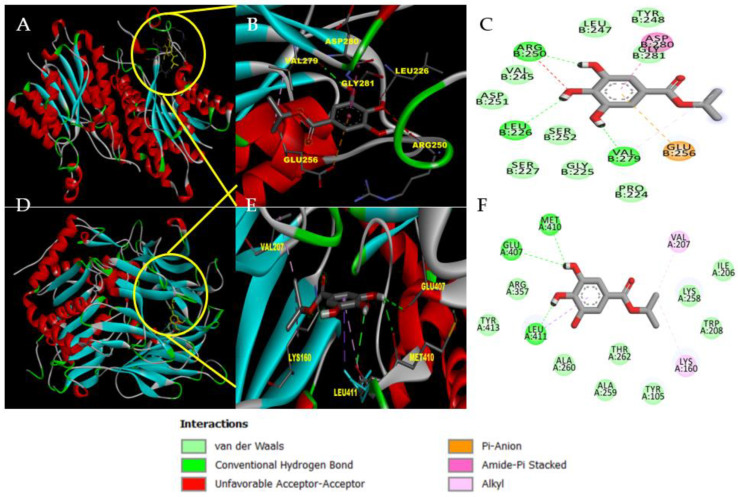
Molecular docking of the most favorable interaction of IPG with the active site of PTR1 and the active site of OPB. (**A**) Active site of pteridine reductase—PTR1; (**B**) 3D interaction of IPG with PTR1; (**C**) 2D interaction of IPG with PTR1; (**D**) Active site of oligopeptidase B—OPB; (**E**) 3D interaction of IPG with OPB; (**F**) 2D interaction of IPG with OPB.

The affinity of PTR1 for IPG can be justified by the presence of some strong bonds in the target–ligand complex, such as hydrogen bonds with the amino acid residues Arg250, Leu226 and Val279. In addition to these, van der Walls bonds were formed with the residues of Pro224, Gly225, Ser227, Ser252, Asp251, Val245, and Gly281 (Figure 3A–C). In turn, molecular docking with OPB formed hydrogen bonds with residues Met410, Glu407, and Leu411, and Van Der Walls bonds with Arg357, Tyr413, Ala260, Ala259, Thr262, Tyr105, Lys258, Trp208, and Ile206 (Figure 3D–F).

Both PTR1 and OPB are important enzymes for leishmania and are therefore promising targets for drug development against this parasite. This is because PTR1 participates in the folate recovery pathway and is unique to the trypanosomatid protozoan family, contributing to the development of selective inhibitors [29]. Furthermore, promastigotes of *L. major* with problems in OPB expression have deficiencies in the transition from procyclic to metacyclic form, considered the infective stage of leishmania, besides considerable loss in the capacity of infection and survival in macrophages [29]. 

With the prediction of physicochemical properties (Table 3), it was possible to characterize the molecule and predict pharmacokinetic parameters. Molecular weight ≤ 500 g/mol, log P ≤ 5, H-bond Donors (HBD), ≤5 and H-bond Acceptors (HBA) ≤ 10 are part of Lipinski’s rules, which were predicted as acceptable for IPG. With this, IPG is a candidate molecule for oral drug [30].

IPG was classified, according to the scale described in the literature [31], as a soluble molecule, which is important in the absorption process, which was significantly high (99.6%) in the prediction model [32]. However, using the model of human colon adenocarcinoma cells (Caco-2), which mimic human intestinal enterocytes [33], IPG showed low intestinal permeability. The difference in response between the human intestinal absorption model and Caco-2 cells is also reported in in vitro studies, and the absence of important transporters in the Caco-2 model is pointed out, which explains why some drugs show low permeability to Caco-2 and yet are well absorbed in the intestine [34]. 

IPG showed a binding percentage to plasma proteins of 76%, presenting a free fraction of 34%, which will be available to exert its pharmacological functions or be transformed into metabolites [35]. It was also observed that IPG may act as a P-glycoprotein inhibitor, which may lead to an increase in drug concentration within cells [36], as already demonstrated for other alkyl gallates such as propyl gallate [37]. 

As for permeability in the central nervous system (CNS), IPG presented within the reference values of 0.1–2.0 for pharmacologically active and inactive compounds in the CNS [38]. In fact, other polyphenols with similar structure, such as epigallocatechin-3-gallate and propyl gallate, can cross the blood–brain barrier [39], which may be favorable in the treatment of leishmaniasis that presents neurological involvement [40]. 

Besides the possibility of oral administration, the IPG showed a value close to the desired value, which varies between −3 and +6, for molecules with possibility of being absorbed by the transdermal route, and this parameter can be improved during the development of a topical formulation [41]. The development of topical formulation would be an alternative for the treatment of LC caused by *L. major* since conventional treatments trigger strong adverse reactions related to the high toxicity of the compounds used and the recommended routes of administration [42].

Regarding hepatic metabolism phase, in silico studies showed that IPG did not inhibit the cytochrome P450 isoenzymes: CYP3A4, CYP1A2, CYP2C19, CYP2C9, and CYP2D6, which limits its biotransformation and possibility of interactions with other drugs, reducing the risks of adverse effects [43]. 

To evaluate the safety in the use of IPG, the analysis shown further in (Table 3) indicates that this molecule is not hepatotoxic and does not inhibit the K+ channels of the human ether-a-go-go related gene (hERG) involved in repolarization of the cardiac action potential, thus reducing the risks of cardiotoxicity [44]. This polyphenol did not show toxicity in acute oral toxicity models, being classified in category III; that is, IPG needs a median lethal dose (LD_50_) between 500 mg/Kg and 5000 mg/Kg to be able to cause toxicity in the body [45]. Additionally, the possibility of eye injury and absence of eye corrosion was observed.

The genomic toxicity analysis revealed that IPG is not mutagenic or carcinogenic. For the parameter of genotoxicity, through the micronucleus test, the IPG was positive. This test is a measure of genetic damage, where it is possible to identify compounds that cause cytogenetic damage resulting in the formation of micronuclei, with the presence of chromosomal fragments or whole chromosomes [46]. In tests of chromosome aberration in CHO-K1 cells (Chinese hamster ovary fibroblast), propyl gallate showed positive results [47]. 

### 3.2. In Vitro Analysis: IPG-Induced Antileismanial Effects and Cell Viability

The evaluation of the antileishmanial activity of IPG showed the inhibition of the growth of promastigotes of *L. major* in all concentrations tested, observing about a 100% inhibition at the concentration of 3770 µM, as shown in (Table 4). The mean inhibitory concentrations (IC_50_) were 90.81 µM for IPG and 1.40 µM for Anf B. Phenolic compounds have exhibited in vitro antileishmanial activity against early stationary phase promastigotes of *L. infantum* and *L. major* [48].

With the MTT assay in macrophages, IPG presented a CC_50_ = 1260 μM (Table 4), and the molecule was considered to have low toxicity in these cells because it did not induce damage to cellular metabolism, as to the activity of mitochondrial dehydrogenases at the concentrations tested. On the other hand, Anf B showed high cytotoxicity on murine macrophages, presenting a CC_50_ of 8.97 μM. These results showed that IPG displayed little cytotoxic effect (CC_50_ = 1470 µM), similar to that which was observed in the MTT test, confirming its safety. Regarding toxicity in sheep erythrocytes, IPG showed no cytotoxic action on these cells, demonstrating a CH_50_ > 3770 µM. A similar result was reported in the literature, which reported that the IPG exhibited lower cytotoxicity against erythrocyte cells [16].

**Figure 4:**
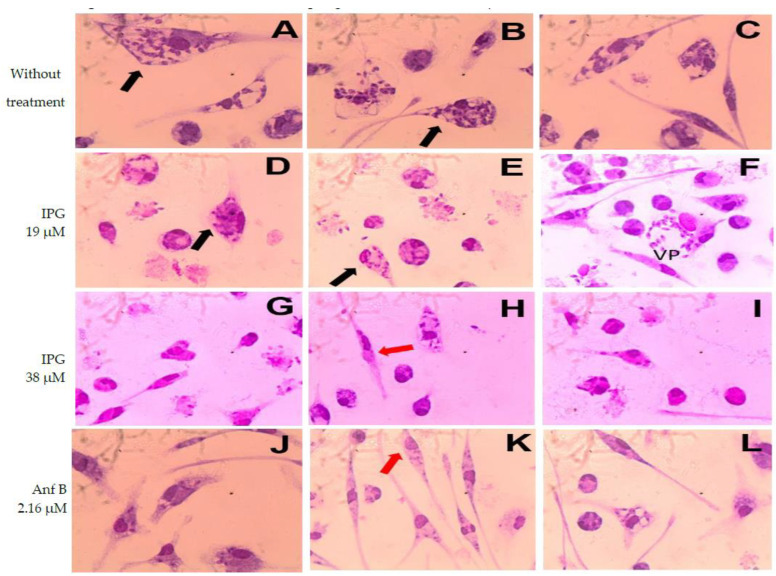
Murine macrophages experimentally infected by *L. major* without treatment (**A**–**C**) and treated with IPG at concentrations of 19 μM (**D**–**F**) and 38 μM (**G**–**I**). Amphotericin B was used as a positive control at the concentration of 2.16 μM (**J**–**L**). VP—Parasitophorous vacuole. Black arrows indicate internalized amastigote forms. Red arrows indicate smeared macrophages. Magnification 1000×.

**Figure 5:**
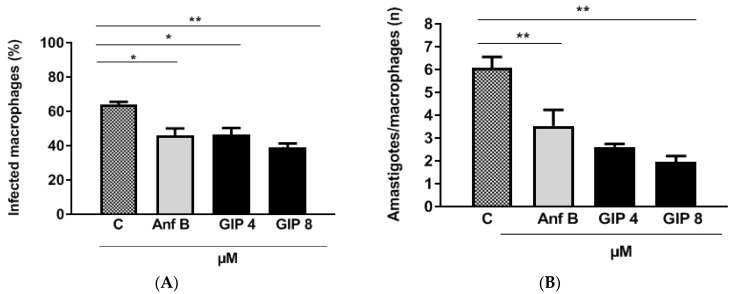
Effects of IPG and Anf B as a reference drug to assess infection (infected macrophages (**A**) and infectivity (**B**) in the treatment of murine macrophages infected with *L. major*. The percentage of infection after treatment (**A**) and the number of amastigotes per macrophage (**B**) were calculated by counting 100 cells in triplicate. Results are presented as mean ± SEM. of three independent experiments performed in triplicate, * *p* < 0.05, ** *p* < 0.01, when compared with control C, Anf B or the concentrations tested.

To verify the effects of IPG on intracellular amastigotes, murine macrophages were infected with *L. major* and treated with IPG and Anf B. The results of the in vitro infection are shown in photomicrographs of these macrophages (Figure 4). The data obtained indicate that IPG was able to reduce the infection and infectivity of infected macrophages (Figure 5A).

IPG showed a better result in the treatment of infection at the concentration of 38 μM, probably because its action is not only through mechanisms that directly affect the parasite, but also because it presents intracellular characteristic changes that are essential to its development. The presence of agglomeration of amastigotes around the parasitophoric vacuole can be observed in F (IPG at 19 μM), the “smoky” aspect of macrophages in F (IPG at 19 μM), G (IPG at 38 μM), H (IPG at 38 μM), I (IPG at 38 μM), and “smeared” appearance of macrophages in H (IPG at 38 μM), which are characteristic of macrophage activation, which makes the cell able to react to and resolve the installed infection [49].

When analyzing infectivity, the negative control obtained an average of six amastigotes/macrophages. Anf B at a concentration of 2.16 μM halved the number of parasites, leading to approximately four amastigotes/macrophages. IPG reduced the amount of amastigote in a concentration-dependent manner, resulting in an average of three amastigote/macrophages when treated at the concentration of 19 μM. When treated at the concentration of 38 μM, this average decreased to two amastigote/macrophages (Figure 5B). The calculated IC_50_ for IPG and Anf B on infectivity were 13.45 μM and 0.90 μM, respectively. Therefore, IPG was shown to be more selective to parasites than to mammalian cells, showing a selectivity index (SI) of 93.8. Anf B proved to be more selective for macrophages than for parasites, with a SI of 9.97 (Table 4).

IPG stood out for its excellent activity against amastigotes, with a value of (IC_50_ = 13.45 μM), approximately seven times lower than the value found for promastigotes (IC_50_ = 90.81 μM). IPG presented IS = 93.8, which suggests that the compound is more selective for the amastigote forms of *L. major* than for murine macrophages when compared to Anf B, a reference drug used in the treatment of leishmaniasis, which displayed a SI of 9.97 [50].

**Figure 6:**
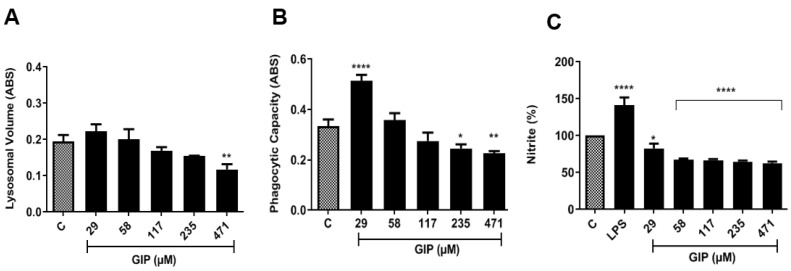
Influence of IPG on lysosomal volume, phagocytic capacity of murine macrophages and nitric oxide. (**A**) (Lysosomal volume); (**B**) (phagocytic capacity), and (**C**) (nitrite) represents the mean ± SEM of three experiments performed in triplicate, being * *p* < 0.05; ** *p* < 0.01; **** *p* < 0.0001. C—control, LPS—*Escherichia coli* lipopolysaccharide.

The results regarding macrophage activation and immunomodulation parameters, such as lysosomal volume and phagocytic capacity, were evaluated as shown in (Figure 6). IPG was able to increase the lysosomal volume of macrophages at the concentration of 29 μM. Additionally, an increase in phagocytic capacity was observed at the concentrations 29 μM and 58 μM. Phagocytic capacity is increased when zymosan stimulates defense cells to induce response; this leads to an increase in both interferon gamma (IFN-γ) production and phagocytic capacity [51]. These data corroborate the literature [11], which reported excellent results regarding macrophage activation parameters, showing that gallic acid and ellagic acid were able to increase lysosomal volume and phagocytic capacity [52]. 

Nitric oxide (NO) production is also a parameter of macrophage activation, which is indirectly quantified by measuring nitrite concentrations by incubating macrophages with IPG. Bacterial lipopolysaccharide from *Escherichia coli* (LPS) was used as a positive control. In relation to the control and LPS, the values found for macrophages treated with IPG demonstrate that there was no significant increase in the NO activation pathway, verifying that leishmanicidal activity does not occur through this pathway [53]. This can be explained by the observation that, at the highest concentrations of IPG, in the absence of stimulation of NO synthesis, a greater toxicity on macrophages was noticed. This can also be associated with the antioxidant effect of IPG.

## 4. Conclusions 

The IPG showed good in silico binding affinity to leishmania targets (PTR1 and OPB), with good lipophilicity and oral bioavailability, as well as good skin penetration within the recommended values, which encourages further studies concerning development of topical formulations for promising applications in experimental models of cutaneous leishmaniasis. Interestingly, IPG showed a significant effect against promastigotes and amastigotes forms of *L. major*, acting selectively against parasite rather than host cells. The very high selectivity index of IPG was markedly higher than that found for Amphotericin B, which supports further studies comprising IPG as a possible novel alternative for counteracting parasitic resistance to antimicrobials, and then opens to the development of novel and effective treatments of leishmaniasis. 

## Figures and Tables

**Table 1:** Main targets for antileishmanial chemotherapy for *Leishmania major* species.

Targets	PDB *	Metabolic Pathway/Function
Nucleoside Hydrolase (NH)	1EZR	Nucleic acid metabolism
Oligopeptidase B (OPB)	2XE4	Protease/virulence factor
Leishmanolysin Proteinase (*Gp63*)	1LML	Virulence factor
Pteridine Reductase (PTR1)	1E7W	Nucleic acid metabolism
Triparedoxin peroxidase (TxP)	3TUE	Redox metabolism (parasite survival)

Legend: * PDB (*Protein Data Bank*): 3D database of proteins and nucleic acids.

**Table 2:** Binding energies of IPG with *L. major* targets.

GIP Molecule	Targets				
Interaction energy (kcal/mol)	NH	OPB	Gp63	PTR1	TxP
	−7.3	−8.0	−6.4	−8.2	−6.0

Legend: Nucleoside hydrolase (NH); oligopeptidase B (OPB); leishmanolysin proteinase (gp63); pteridine reductase (PTR1); triparedoxin peroxidase (TxP).

**Table 3:** In silico ADMET properties of compound IPG.

Properties	Parameter	Prediction
	Molecular Weight (g/mol)	212.2
	Log of lipophilicity (Log P)	1.37
Physical Chemistry	Aqueous solubility (Log S)	−2.22
	H-bond donors (HBD)	3
	H-Bond Acceptors (HBA)	5
	Human oral bioavailability (%)	0.5429
	Human intestinal absorption (%)	0.9963
	Caco-2 permeability coefficient (nm/s)	0.5405
	Plasma protein binding (PPB) (%)	76.0
	Inhibition of P-glycoprotein (gp-P)	Yes
Pharmacokinetics	Penetration of the blood–brain barrier	0.8301
	Skin permeability	−3.3007
	CYP2C19 inhibitor	No
	CYP2C9 inhibitor	No
	CYP1A2 inhibitor	No
	CYP2D6 inhibitor	No
	CYP3A4 inhibitor	No
	Organ Toxicity	
	Drug-induced liver-injury assay Human ether-a-go-go gene (hERG) K^+^ channels	-
Toxicological	Acute oral toxicity *	III
	Eye injuries	+
	Eye corrosion	-
	Genomic toxicity	
	Ames mutagenesis	-
	Carcinogenesis	-
	Micronucleus assay	+

Legend: Log lipophilicity (Log P); aqueous solubility (Log S); hydrogen bond donors (HBD); hydrogen bond acceptors (HBA); binding to plasma proteins (PPB); P-glycoprotein (gp-P); cytochrome P450 isoenzymes (CYP2C19, CYP2C9, CYP1A2, CYP2D6, CYP3A4); + (toxic); - (non-toxic); * acute oral toxicity level category—category I and II (toxic compound) and category III and IV (non-toxic compound), based on U.S. Environmental Protection Agency (EPA) criteria.

**Table 4:** In vitro antileishmanial activity, cytotoxicity, selectivity index.

Substance	Macrophage CC_50_ (μM)	Erythrocyte CH_50_ (μM)	Promastigote IC_50_ (μM)	Amastigote IC_50_ (μM)	IS_m_	IS_e_
IPG	1260 ± 0.48	>3770	90.81 ± 0.51	13.45 ± 0.35	93.8	>280.3
Anf B	8.97 ± 0.02	ND *	1.40 ± 0.09	0.90 ± 0.36	9.97	ND *

Legend: IS_m_ (amastigote selectivity index) = CC_50_/CI_50_; IS_e_ (erythrocyte selectivity index) = CH_50_/CI_50_; * ND (not determined).

## Data Availability

Not applicable.
